# The effects of the heavy metals cadmium and lead on six metabolic and immune-related enzymes in the loach *(Misgurnus anguillicaudatus)*

**DOI:** 10.7717/peerj.20547

**Published:** 2026-01-07

**Authors:** Qin Wang, Jiejie Su, Zhiruo Fu, Yujia Hu, Junlong Wei, Tao Pan, Shengzhou Zhang

**Affiliations:** 1 College of Life Sciences, Anhui Normal University, Wuhu, Anhui, China; 2 School of Health Science and Engineering, Ma’anshan University, Ma’anshan, Anhui, China

**Keywords:** *Misgurnus anguillicaudatus*, Cadmium, Lead, Metabolism, Immunity, Enzyme, Histochemistry

## Abstract

**Background:**

Cadmium (Cd^2+^) and lead (Pb^2+^) are two common heavy metal pollutants in rivers and lakes that have multiple toxic effects on organisms. However, their toxic mechanisms are not fully understood. The loach (*Misgurnus anguillicaudatus*), belonging to the genus *Misgurnus* (Cypriniformes, Cobitidae), is an important benthic fish species whose physiological activities are highly susceptible to heavy metal pollutants. Such exposure can adversely affect its growth, development, and reproduction, leading to disease occurrence and significant economic losses in the *M. anguillicaudatus* farming industry.

**Methods:**

After *M. anguillicaudatus* was exposed to Cd^2+^ (3.625 mg L^−1^, 7.25 mg L^−1^, and 14.5 mg L^−1^) and Pb^2+^ (47.5 mg L^−1^, 95 mg L^−1^, and 190 mg L^−1^) for 96 h, frozen sections of their visceral organs (heart, hepatopancreas, gills, kidneys, stomach, and intestine) were prepared. The activities of six metabolic and immune-related enzymes in these organs were assessed using enzyme histochemical staining and optical density quantitative analysis technology.

**Results:**

The distribution of the six metabolic and immune-related enzymes exhibited significant tissue specificity. Acid phosphatase (ACP) was mainly distributed in the stomach, intestine, and gills; alkaline phosphatase (ALP) exhibited high activity in the stomach and intestine; and adenosine triphosphatase (ATPase) displayed greater activity in the heart, hepatopancreas, and stomach. In addition, non-specific esterase (NSE) was abundant in the hepatopancreas, stomach, gills, and kidney; peroxidase (POX) activity was prominent in the kidney, gills, and heart; and succinate dehydrogenase (SDH) activity was enriched in the heart and hepatopancreas. Exposure to Cd^2 +^ markedly inhibited ACP activity in all six organs, significantly inhibiting ALP activity in the hepatopancreas, gills, and stomach, ATPase activity in all six organs, NSE activity in the hepatopancreas and stomach, POX activity in the heart, gills, and kidney, and SDH activity in the heart, hepatopancreas, and stomach. Similarly, Pb^2 +^ exposure significantly inhibited ACP and ATPase activities in all organs except the kidney and stomach, the ALP and NSE activities of the hepatopancreas, gills, and stomach, the POX activities of the heart, gills, and kidney, and the SDH activities of the heart, hepatopancreas, and stomach.

**Conclusions:**

Compared with Pb^2+^, Cd^2+^ exerted a broader toxic effect across the six organs. Both heavy metal ions disrupted the blood circulation of *M. anguillicaudatus* by inhibiting enzymatic activity, impairing detoxification and respiration, and reducing the digestion and absorption of nutrients.

## Introduction

Heavy metal pollution has become an increasingly prominent environmental problem mainly due to industrial wastewater and waste slag from the mining industry (Gabriel et al., 2020; Sharma et al., 2024b). Heavy metals are non-degradable and bioaccumulate in aquatic ecosystems. They can enter aquatic organisms through various channels, such as feeding, breathing, and skin contact (Garai et al., 2021; Moiseenko & Gashkina, 2020; Muneer et al., 2021). In organisms, heavy metals are not only enriched and accumulated, but may also undergo biomagnification, with their concentration increasing gradually through the food chain. This stepwise transfer ultimately poses serious risks to aquatic organisms, aquatic ecosystems, and human health (Devi, 2024; Saravanan et al., 2024; Sharma et al., 2024a). Numerous studies have shown that heavy metal toxicity can lead to DNA damage, protein destruction, decreased enzyme activity, and apoptosis. Thus, it damages the physiological activities of aquatic organisms, such as embryonic development, growth, reproduction, and feeding (Li et al., 2022; Su et al., 2023; Waqas et al., 2024).

Cadmium (Cd^2+^) and lead (Pb^2+^) are among the most common heavy metal pollutants in the sediments of the lower mainstream of the Yangtze River, China, and are also prevalent toxic elements in many rivers and lakes worldwide (Hossain et al., 2021; Jin et al., 2024; Muneer et al., 2021). It has been reported that the dissolved concentrations of Cd^2+^ and Pb^2+^ in the polluted waters of the Hengshi River, China, are 0.103 and 2.91 mg/L, respectively (Zhou et al., 2005). In addition, studies have shown that the concentration of Pb^2+^ in surface water of the Aliaǧ a metal industry zone in Turkey is as high as 1.52 ± 0.3 mg/L (Sponza & Karaoǧlu, 2002). In recent years, there have been many reports on the toxic effects of Cd^2+^ and Pb^2+^ on aquatic life. For instance, Hu & Wang (2024) showed that Cd^2+^ exposure inhibits the skeletal mineralization of zebrafish (*Danio rerio*) and aggravates motor behavior disorders. Cd^2+^ exposure also reduces the success rate of paired egg laying in rare minnow (*Gobiocypris rarus*) parents, prolongs the first egg laying time, and causes reproductive problems, such as follicle dysplasia and sperm vacuolization (Su et al., 2023). Cd^2+^ exposure disrupts the energy balance of shrimp (*Litopenaeus vannamei*), resulting in reduced metabolism and ammonia excretion and increased mortality (Da Costa et al., 2024).

Pb^2+^ exposure can lead to liver and kidney damage in African catfish (*Clarias gariepinus*) and changes in serum biochemical parameters (Onadje & Akalusi, 2024). Pb^2+^ exposure can also lead to decreased survival, delayed hatching, abnormal movement frequency and heart rate, and spinal malformations in zebrafish embryos (Li et al., 2022). Therefore, Cd^2+^ and Pb^2+^ have many toxic effects on different organisms. Nevertheless, research on the mechanisms underlying their toxicity remains limited, and further research is needed.

Enzymes are catalytic biomolecules involved in various metabolic reactions within organisms. Most enzymes are proteins and are highly sensitive to environmental changes, especially to heavy metal exposure (Shahjahan et al., 2022). The heavy metal zinc (Zn^2+^) has been shown to inhibit gill and renal Na^+^/K^+^-ATPase activity in rainbow trout (*Oncorhynchus mykiss*) (Haverinen & Vornanen, 2023), while manganese (Mn^2+^) and nickel (Ni^2+^) can inhibit glutathione reductase activity in the liver of scorpionfish (*Scorpaena porcus*) (Işık & Soydan, 2023). In addition, heavy metal exposure can lead to increased levels of serum and antioxidant enzymes, such as aspartate aminotransferase, alanine aminotransferase, superoxide dismutase, and glutathione peroxidase (GP_X_) (Liu et al., 2022; Shahjahan et al., 2022). Therefore, enzymes can be used as valuable indicators for evaluating the mechanisms of pollutant-induced biological toxicity (Ali et al., 2021), and changes in their activities can provide deeper insights into the toxic effects of heavy metals.

*Misgurnus anguillicaudatus*, belonging to the Cobitidae family and Cypriniformes order, is widely distributed across China, Japan, North Korea, Russia, and India. It is highly valued by consumers because of its delicious meat and high nutritional value. In China, the aquaculture production of *M. anguillicaudatus* reached 374,981 t in 2022 and continues to increase (Zhao et al., 2025). However, during the breeding process of *M. anguillicaudatus*, mortality often increases due to water pollution, causing significant economic losses. To evaluate the effects of the heavy metals Cd^2+^ and Pb^2+^ on the health of *M. anguillicaudatus*, six important metabolism and immune-related enzymes were investigated in this study: acid phosphatase (ACP), alkaline phosphatase (ALP), adenosine triphosphatase (ATPase), peroxidase (POX), non-specific esterase (NSE), and succinate dehydrogenase (SDH). These six enzymes play central roles in key physiological processes, such as glucose metabolism, energy conversion, detoxification, and immune defense (Atli et al., 2013; George et al., 2011; He et al., 2016; Kinareikina, Silivanova & Kyrov, 2024; Marenkov et al., 2021). Changes in their activities can reflect the response of organisms to heavy metal stress with high sensitivity, making them reliable biomarkers for evaluating the metabolism and immune functions of *M. anguillicaudatus.* To the best of our knowledge, this is the first study to investigate the effects of Cd^2+^ and Pb^2+^ on the activities of six key enzymes in major organs of *M. anguillicaudatus* using enzyme histochemical staining and quantitative analysis of optical density. Furthermore, the biological toxic effects of Cd^2+^ and Pb^2+^ on *M. anguillicaudatus* were explored. These findings enhance our understanding of the mechanisms underlying Cd^2+^ and Pb^2+^ toxicity and provide basic information for the environmentally sustainable breeding of *M. anguillicaudatus*.

## Methods

### Experimental animals

A total of 360 healthy adult *M. anguillicaudatus* were cultured, with an average body length of 15.5–18.5 cm and a body weight of 25–35 g. The selected individuals had no scars, exhibited normal swimming behavior, and showed no signs of disease. Both males and females were included, with no significant difference in enzyme activity between the sexes. From March to September 2023, the *M. anguillicaudatus* were purchased from local farms in Wuhu City. After purchase, they were acclimated in the laboratory’s breeding tanks for one week. The width, height, and length of each tank were 52 cm, 37 cm, and 32 cm, respectively. Nine *M. anguillicaudatus* individuals were placed in each tank, and the water was replaced every 24 h. During this period, natural light was used, feed was supplied regularly, and no deaths occurred. The static aquaculture system used tap water that had been aerated for one week, with a pH of 7.2–7.5 and a water temperature of around 25 °C. Animal care, housing, and feeding were conducted in accordance with animal ethics guidelines. For sampling purposes, the *M. anguillicaudatus* individuals were euthanized with tricaine methanesulfonate (MS-222). At the conclusion of the experiment, all remaining *M. anguillicaudatus* were euthanized humanely, and no humane endpoints were utilized in this study. This procedure was approved by the ethics committee of Anhui Normal University (approval no: AHNU-ET2025068). Fish handling and sampling techniques were conducted in accordance with standard vertebrate procedures, veterinary practices, and national and provincial guidelines.

### Treatment of *M. anguillicaudatus*

Based on preliminary experiments, the 50% lethal concentration (LC_50_) of Cd^2+^ for *M. anguillicaudatus* was 28.96 mg/L, while that of Pb^2+^ was 380.3 mg/L. The LC_50_/8, LC_50_/4, and LC_50_/2 were used as the toxic concentrations of Cd^2+^ and Pb^2+^ for determining the effects on the activities of six important enzymes. Accordingly, three cadmium chloride (CdCl_2_) toxic concentrations were 3.625 mg L^−1^, 7.25 mg L^−1^, and 14.5 mg L^−1^, and lead nitrate (Pb(NO_3_)_2_) toxic concentrations were 47.5 mg L^−1^, 95 mg L^−1^, and 190 mg L^−1^. The water quality parameters of the tap water used in the exposure process were as follows: a temperature of 25 ± 1.0 °C, pH 7.0–7.1, and a hardness of 142.6 mg/L. A control group without heavy metals was included, and each treatment was replicated five times. For exposure, 10 L of the prepared CdCl_2_ and Pb(NO_3_)_2_ solution were added to each breeding tanks. Healthy and energetic *M. anguillicaudatus* were selected and randomly assigned into four groups (nine in each group) using a computer-generated randomization table, and exposed for 96 h.

### Cryosection preparation

After the toxicity exposure experiments, the *M. anguillicaudatus* individuals were euthanized *via* prolonged immersion in an overdose of MS-222 (Fujian Jinjiang Shengyuan Aquatic Products Co., Ltd., Jinjiang, China), in accordance with the American Veterinary Medical Association animal euthanasia guidelines. Immediately after euthanasia, the fish were dissected and the heart, liver, gills, kidney, stomach, and intestine were excised and quickly rinsed with phosphate-buffered saline (PBS, pH 7.4). The surface moisture was absorbed with drying paper, and the tissues embedded in an embedding medium (OCT). Tissue sections were prepared at −26 °C using a cryostat (CM 1900, Leica, Wetzlar, Germany) at a thickness of 8 µm and temporarily stored at −20 °C.

### Enzyme histochemical staining

Enzyme histochemical staining was performed according to the methods described by Xie et al. (2016), with slight modifications.

#### Acid phosphatase staining

The Pb^2+^ method was used for color development, and 15 mL of 0.2 mol/L acetic acid buffer solution (pH 5.2), 15 mL of 0.24% Pb(NO_3_)_2_ solution, and three mL of 3% *β*-glycerol sodium phosphate solution were mixed thoroughly and placed in a 37 °C water bath for 25 min. This mixture was filtered to obtain the incubation solution, which was stored in a refrigerator at 4 °C until further use. The prepared frozen tissue sections were placed in a wet box, and the incubation solution was added dropwise onto each section. Next, the sections were incubated in a constant temperature chamber at 37 °C for 40 min, followed by rinsing with distilled water. After drying, 2% ammonium sulfide solution was added and allowed to react for 2 min. Finally, the sections were rinsed with distilled water and sealed with gum after drying.

#### Alkaline phosphatase staining

Color development was performed using the nitro blue tetrazolium (NBT)/5-bromo-4-chloro-3-indolyl-phosphate (BCIP) method. Briefly, 9.9 µL of 5% NBT solution was mixed with 1.5 mL of 0.1 mol/L tris-buffered saline (TBS) buffer, and then 4.95 µL of 5% BCIP solution was added to prepare the incubation solution, which was then used immediately. The solution was added dropwise to the frozen sections and allowed to react at room temperature for 20 min. The sections were then rinsed with distilled water and sealed with neutral gum after drying.

#### Adenosine triphosphatase *staining*

The calcium-cobalt method was used for color development. Briefly, 15 mg of disodium ATP (ATP sodium salt) was dissolved in four mL of distilled water, followed by the addition of two mL of 2% sodium barbital, one mL of 2% anhydrous calcium chloride, and five mg of 2,4-dinitrophenol. Next, seven mL of distilled water was added to prepare the incubation solution, the pH was adjusted to 9, and the solution was incubated in a 37 °C water bath for 15 min, filtered, and stored at 4 °C. Prior to incubation, frozen sections were pretreated with 1% CaCl_2_ solution at 37 °C for 5 min. After incubation, the CaCl_2_ solution was aspirated with absorbent paper, and the already configured incubation liquid was added to the sections. Next, they were placed in a wet box in a constant temperature box at 37 °C for 90 min and rinsed with distilled water. After drying, 2% cobalt nitrate solution was added for 5 min, followed by rinsing with distilled water and fixation with 4% neutral formaldehyde for 2 min. The sections were then rinsed again with distilled water, stained with 2% ammonium sulfide solution for 2 min, washed with distilled water, and then sealed with gum after drying.

#### Non-specific esterase *staining*

Color development was performed using the azo method. Briefly, 0.4 g of alkaline magenta powder was dissolved in 0.6 ml of concentrated hydrochloric acid, and rapidly stirred with a glass rod to form a brown-purple paste. Subsequently, eight mL of distilled water was added, and the solution was filtered and mixed with an equal volume of 4% NaNO_2_ aqueous solution to prepare a hexazo sub-magenta red solution. Separately, 0.01 g of 1-naphthol acetic acid was dissolved in 0.4 mL of acetone, followed by addition of 40 µL of PBS and 2.4 mL hexazo by-maru red. The resulting solution was added dropwise onto the frozen sections and incubated at room temperature for 30 min. The sections were then rinsed with distilled water, air-dried, and sealed with gum.

#### Peroxidase *staining*

The diaminobenzidine (DAB) method was used for color development. One tube of DAB was dissolved in one mL of 0.1 mol/L TBS buffer, followed by the addition of 3 µL of 36% H_2_O_2_. The solution was mixed thoroughly and applied dropwise to the tissue sections before color development began. Next, sections were stained in a 37 °C incubator for 30 min, rinsed with distilled water, and air-dried at room temperature for 5 min. Finally, 0.1 mol/L PBS was added, rinsed again with distilled water, dried, and sealed with gum.

#### Succinate dehydrogenase *staining*

Color development was conducted using the tetrazole salt method. Briefly, 15 µL of 5% NBT solution was pipetted with a 20 µL pipette and mixed with 1.5 mL of PBS (pH = 7.4). Next, 0.03 g of sodium succinate was added to prepare an incubation solution. The sections were placed in a wet box and after drying, the incubation solution was applied dropwise onto the sections. The sections were incubated in a constant temperature chamber at 37 °C for 30 min, rinsed with distilled water, dried, and sealed with gum.

### Optical density measurement

The sections were observed and photographed at 200× magnification using an Olympus BX61 microscope equipped with an Olympus DP71 digital camera coupled to a microcomputer system (Tokyo, Japan). The captured images were analyzed using Image-Pro Plus software (Media Cybernetics Inc., Rockville, MD, USA) for optical density analysis of the positive parts of the histochemical staining of six enzymes in the six selected organs. The mean optical density (MOD) was calculated using the following formula:

\begin{eqnarray*}\text{MOD}=\text{IOD}/\text{area} \end{eqnarray*}

where the IOD is the integrated optical density, and the MOD was used to represent the enzyme activity strength.

### Statistical analysis

All experimental data were expressed as the mean ± standard deviation. The IBM SPSS Statistics 19 software was used for the two-tailed independent Student’s *t*-test (SPSS Inc, Chicago, IL, USA), and GraphPad Prism 8 software (GraphPad Software Inc, La Jolla, CA, USA) was used to display the data as histograms to determine significant differences in the MOD value between the experimental group and the control group. A significant difference was defined as *P* <  0.05, and *P* < 0.01 was considered highly significant. No outlier tests were conducted, and no data were excluded from the analysis.

## Results

### Distribution of six essential enzymes in *M. anguillicaudatus*

**Figure 1:**
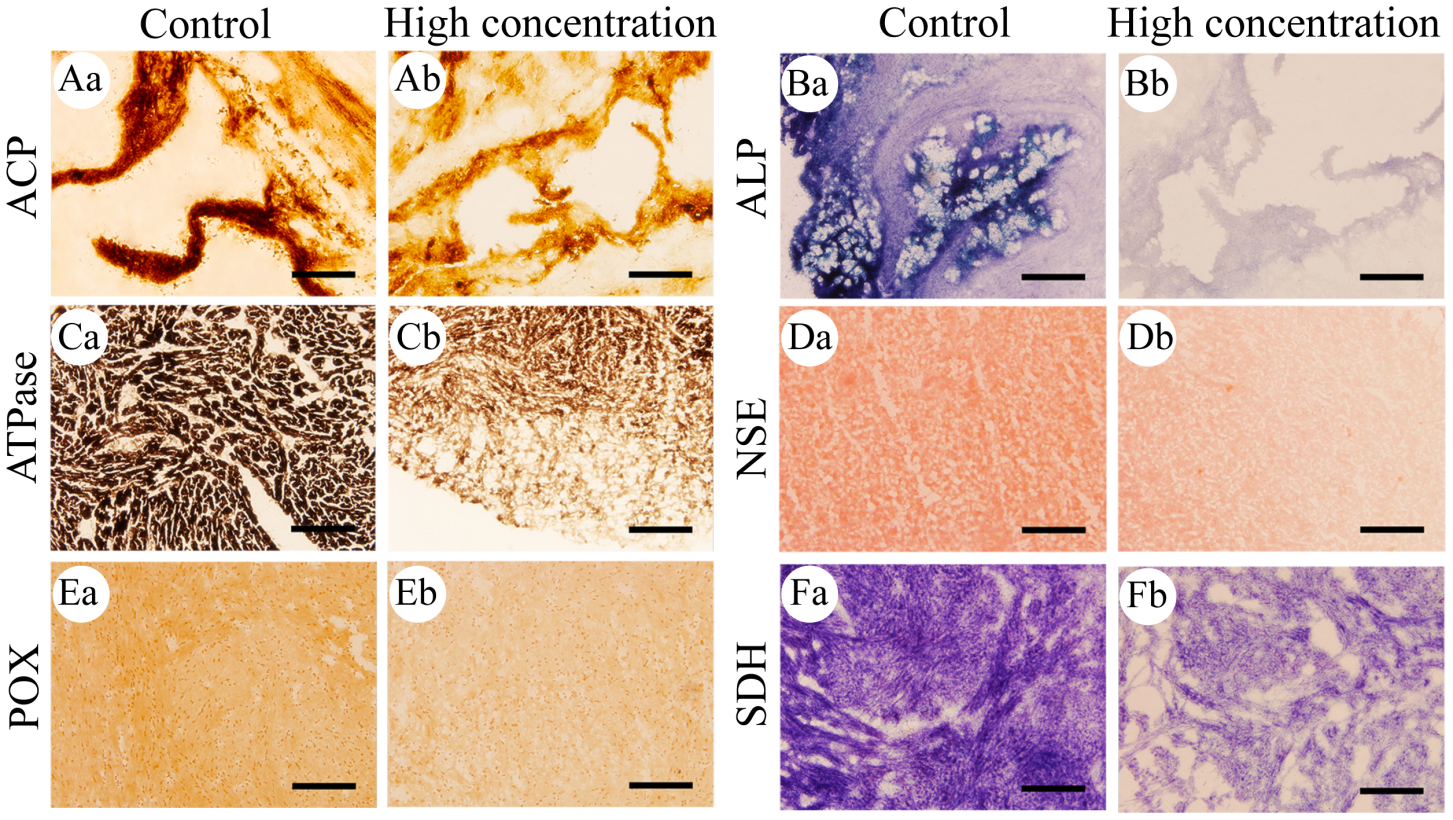
Histochemical examination of the effects of Cd^2+^ on the activities of six metabolic and immune-related enzymes in *Misgurnus anguillicaudatus*. ACP, acid phosphatase; ALP, alkaline phosphatase; ATPase, adenosine triphosphatase; NSE, non-specific esterase; POX, peroxidase; SDH, succinate dehydrogenase. (A) stomach, (B) stomach, (C) heart, (D) hepatopancreas, (E) heart, and (F) heart. Lowercase letters denote control (a) and high concentration of Cd^2+^(b). Aa and Ab, Stomach; Ba and Bb, Stomach; Ca and Cb, Heart; Da and Db, Hepatopancreas; Ea and Eb, Heart; Fa and Fb, Heart. Scale bars = 50 µm. The magnification is 200×.

The histochemical staining of the six important enzymes showed distinct color reactions. Based on quantitative analysis of the optical density, the relative activities of the six enzymes in different parts of the *M. anguillicaudatus* body are summarized in Table 1. Histochemical staining of ACP appeared brown-black (Fig. 1Aa), with the highest enzyme activity located in the stomach and intestine, followed by the gills, hepatopancreas, and kidney, while the heart exhibited the lowest activity. ALP staining appeared blue-purple (Fig. 1Ba), with the highest enzyme activity located in the stomach, followed by the intestine and hepatopancreas, and the lowest activity in the gills. Histochemical staining of ATPase appeared dark in color (Fig. 1Ca), with the highest enzyme activity in the heart, followed by the hepatopancreas, stomach, gills, and intestine, while the kidney showed the lowest activity. NSE staining appeared brick red (Fig. 1Da), with the highest enzyme activity in the hepatopancreas, followed by the stomach and gills, and the kidney and intestine, while the enzyme activity was the lowest in the heart. The histochemical staining of POX appeared brown-yellow (Fig. 1Ea), with the highest enzyme activity in the kidney, followed by the gills, heart, and hepatopancreas, while the stomach and intestine exhibited the lowest activity. Additionally, the histochemical staining of SDH appeared purple (Fig. 1Fa), with the highest enzyme activity in the heart, followed by the hepatopancreas and stomach, while the enzyme activity was the lowest in the intestine.

**Table 1:** Distribution of six metabolic and immune-related enzymes in *Misgurnus anguillicaudatus* (Mean ± SD, *N* = 18).

Organ types	ACP	ALP	ATPase	NSE	POX	SDH
Heart	0.16 ± 0.03^e^	ND	0.86 ± 0.06^a^	0.10 ± 0.01^f^	0.20 ± 0.02^c^	0.37 ± 0.03^a^
Hepatopancreas	0.38 ± 0.04^c^	0.15 ± 0.01^b^	0.67 ± 0.05^b^	0.22 ± 0.02^a^	0.17 ± 0.03^d^	0.17 ± 0.03^b^
Gill	0.42 ± 0.04^b^	0.13 ± 0.01^c^	0.52 ± 0.03^d^	0.14 ± 0.02^c^	0.22 ± 0.03^b^	ND
Kidney	0.31 ± 0.04^d^	ND	0.25 ± 0.04^f^	0.13 ± 0.01^d^	0.60 ± 0.04^a^	ND
Stomach	0.55 ± 0.04^a^	0.42 ± 0.04^a^	0.64 ± 0.03^c^	0.19 ± 0.02^b^	0.14 ± 0.01^e^	0.13 ± 0.02^c^
Intestine	0.53 ± 0.04^a^	0.16 ± 0.01^b^	0.36 ± 0.03^e^	0.12 ± 0.01^e^	0.13 ± 0.02^e^	0.11 ± 0.02^d^

**Notes.**

ACP acid phosphatase ALP alkaline phosphatase ATPase adenosine triphosphatase ND Not detectable NSE non-specific esterase POX peroxidase SD standard deviation SDH succinate dehydrogenase

Two-tailed independent Student’s *t*-tests were employed for statistical comparisons. Values sharing the same letter (a, b, c, d, e, f) within a column indicate no significant difference (*p* > 0.05).

### The effects of Cd^2+^ on the activities of six important enzymes in *M. anguillicaudatus*

**Figure 2:**
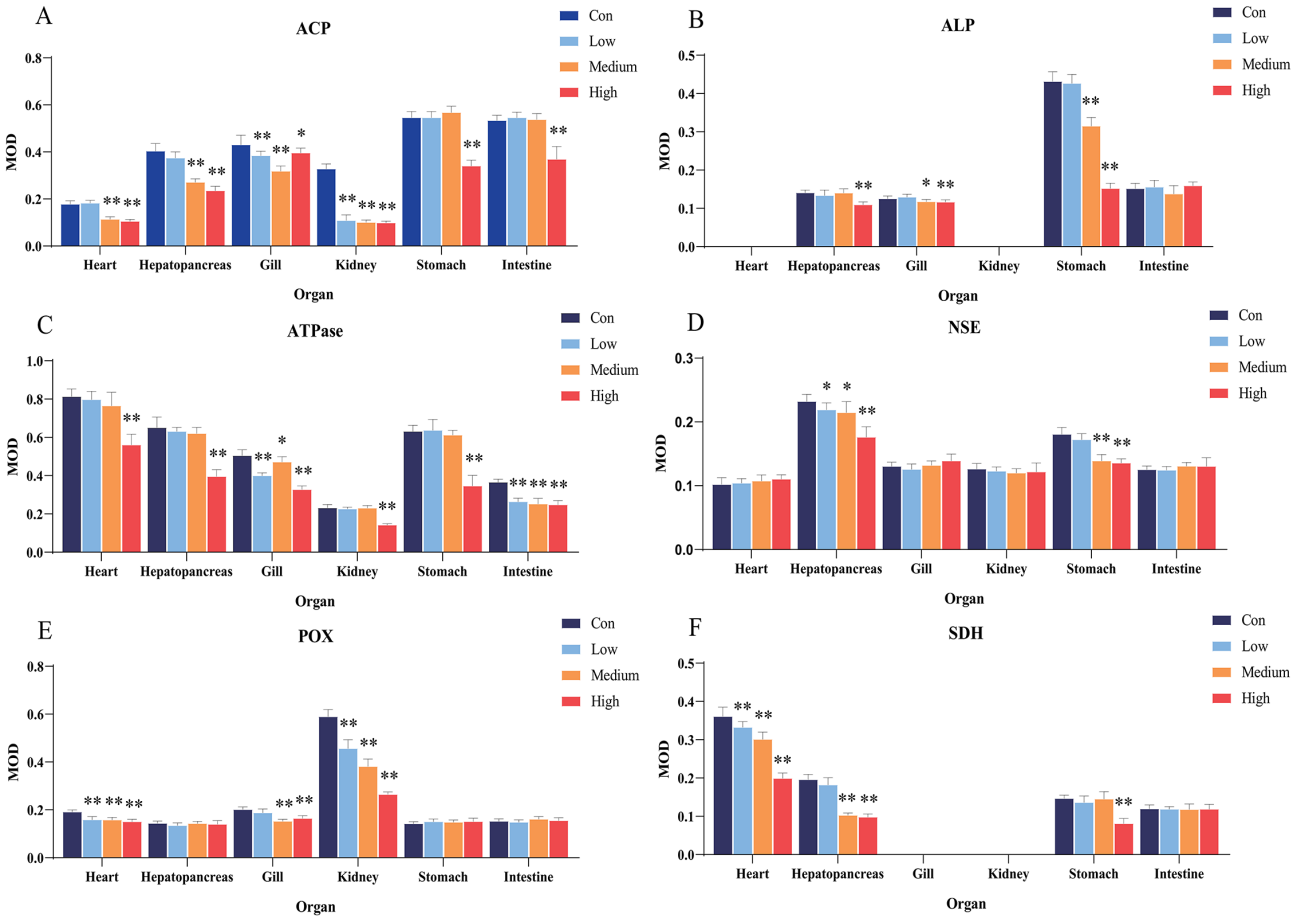
Statistical analysis of the effects of Cd^2+^ on the activities of metabolic and immune-related enzymes in *Misgurnus anguillicaudatus*. ACP, acid phosphatase; ALP, alkaline phosphatase; ATPase, adenosine triphosphatase; MOD, mean optical density; NSE, non-specific esterase; POX, peroxidase; SD, standard deviation; SDH, succinate dehydrogenase. (A), ACP; (B), ALP; (C), ATPase; (D), NSE; (E), POX; (F), SDH. Data are expressed as mean ±  SD, *n* = 9; *significant difference (*p* < 0.05); **extremely significant difference (*p* < 0.01).

After 96 h of Cd^2+^ exposure, the color intensity of the histochemical staining of the six important enzymes in each visceral organ changed significantly (Fig. 1), indicating that the enzyme activity was significantly inhibited. Optical density analysis showed that compared with the control group, Cd^2+^ exposure significantly inhibited the ACP activity in the gills and kidneys at low, medium, and high concentrations. At medium and high concentrations, it significantly inhibited the ACP activity in the heart and hepatopancreas, while at high concentrations, a significant decrease was observed in the stomach and intestine (Fig. 2A).

Compared with the control group, Cd^2+^exposure had no significant effect on ALP activity in any organ at low concentrations. However, it significantly inhibited ALP activity in the gills and stomach at medium and high concentrations, and in the hepatopancreas at high concentrations (Fig. 2B). Compared with the control group, Cd^2+^ significantly inhibited the activity of gill and intestine ATPase at low, medium, and high concentrations, and at high concentrations, the activity was significantly inhibited in the heart, hepatopancreas, kidney, and stomach (Fig. 2C).

In comparison with the control group, Cd^2+^ exposure significantly inhibited hepatopancreas NSE activity at low, medium, and high concentrations, and significantly inhibited stomach NSE activity at medium and high concentrations (Fig. 2D). Similarly, Cd^2+^ exposure significantly inhibited POX activity in the heart and kidney at all concentrations, and in the gills at medium and high concentrations (Fig. 2E). In addition, Cd^2+^ significantly inhibited heart SDH activity at all tested concentrations, significantly inhibited hepatopancreas SDH activity at medium and high concentrations, and significantly inhibited stomach SDH activity at high concentrations (Fig. 2F).

### The effects of Pb^2+^ on the activities of six important enzymes in *M. anguillicaudatus*

**Figure 3:**
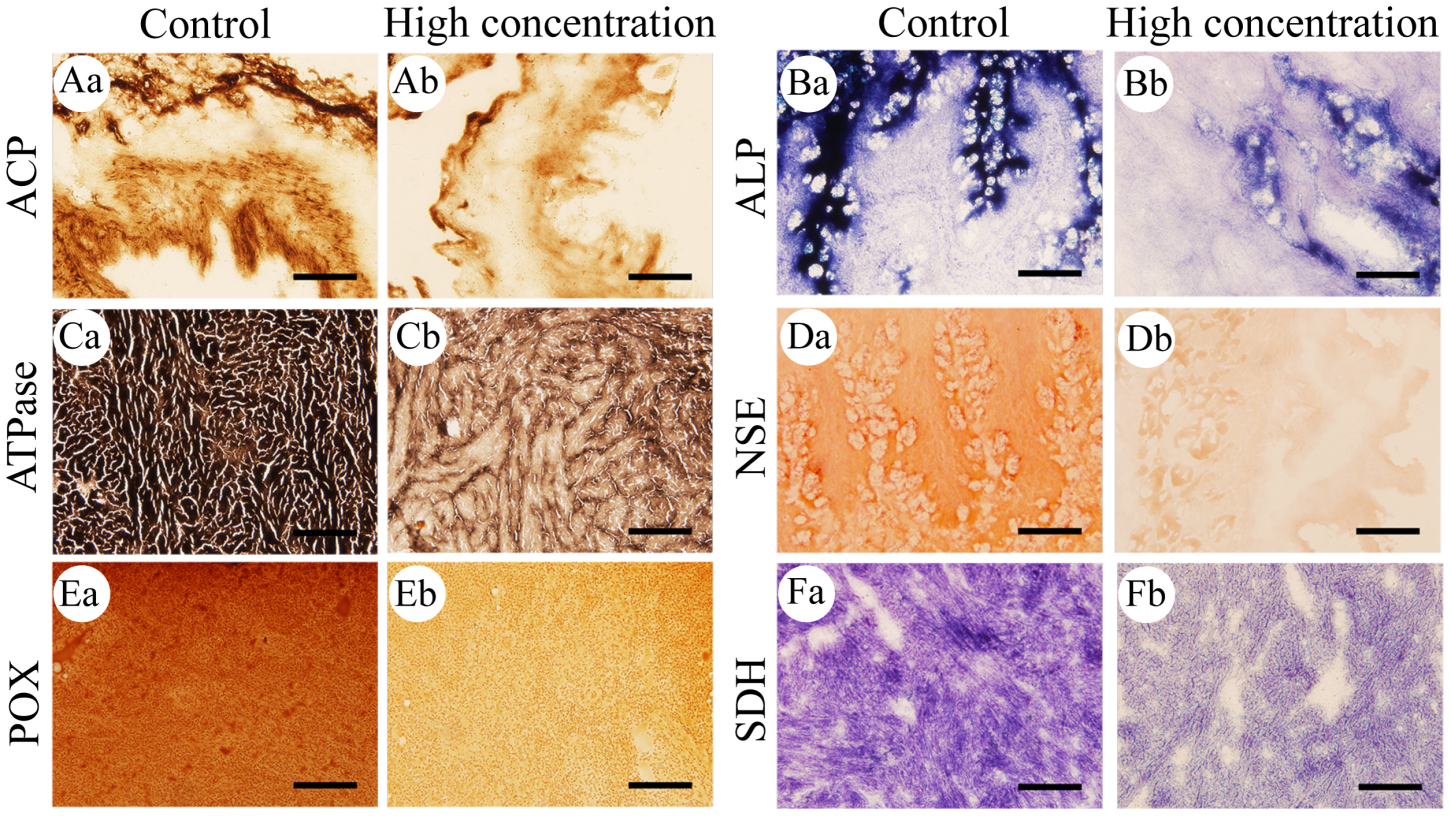
Histochemical examination of the effects of Pb^2+^ on the activities of six metabolic and immune-related enzymes in *Misgurnus anguillicaudatus*. ACP, acid phosphatase; ALP, alkaline phosphatase; ATPase, adenosine triphosphatase; NSE, non-specific esterase; POX, peroxidase; SDH, succinate dehydrogenase. (A) intestine, (B) stomach, (C) heart, (D) stomach, (E) kidney, and (F) heart. Lowercase letters denote control (a) and high concentration of Pb^2+^ (b). Aa and Ab, Intestine; Ba and Bb, Stomach; Ca and Cb, Heart; Da and Db, Stomach; Ea and Eb, Kidney; Fa and Fb, Heart. Scale bars = 50 µm. The magnification is 200×.

**Figure 4:**
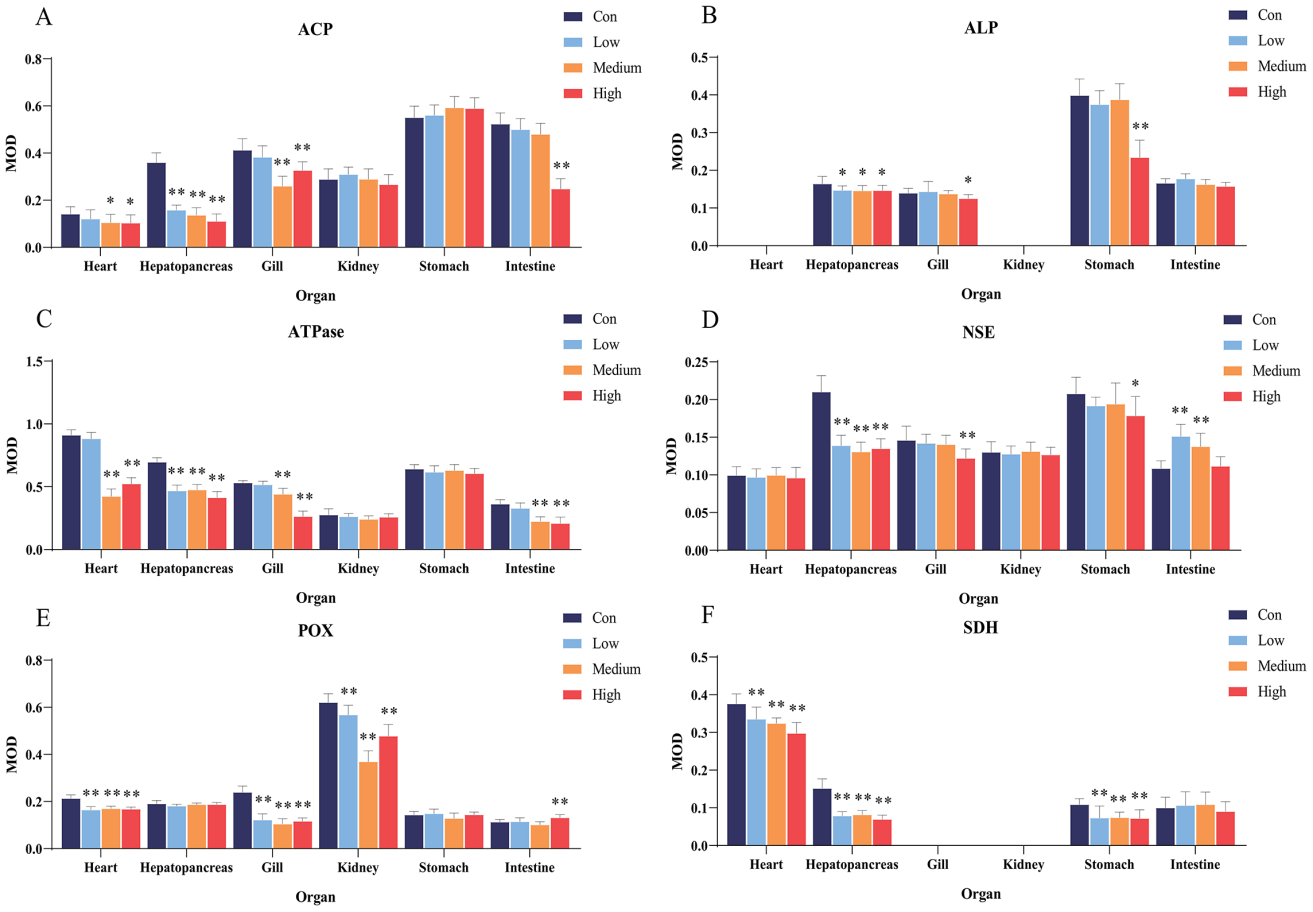
Statistical analysis of the effects of Pb^2+^ on the activities of metabolic and immune-related enzymes in *Misgurnus anguillicaudatus*. ACP, acid phosphatase; ALP, alkaline phosphatase; ATPase, adenosine triphosphatase; MOD, mean optical density; NSE, non-specific esterase; POX, peroxidase; SD, standard deviation; SDH, succinate dehydrogenase. (A), ACP; (B), ALP; (C), ATPase; (D), NSE; (E), POX; (F), SDH. Data are expressed as mean ±  SD, *n* = 9; * significant difference (*p* < 0.05); ** extremely significant difference (*p* < 0.01).

After 96 h of Pb^2+^ exposure, the color intensity of histochemical staining of the six key enzymes in various visceral organs changed markedly (Fig. 3), indicating a significant inhibition of enzyme activity. Optical density statistical analysis showed that, compared with the control group, Pb^2+^ exposure significantly inhibited the activity of hepatopancreas ACP at low, medium, and high concentrations. At medium and high concentrations, ACP activity was also significantly inhibited in the heart and gills, while at high concentrations, it was significantly inhibited in the intestine (Fig. 4A).

Compared with the control group, Pb^2+^ exposure significantly inhibited ALP activity in the hepatopancreas at low, medium, and high concentrations. At high concentrations, it was also significantly inhibited in the gills and stomach (Fig. 4B). Similarly, Pb^2+^ significantly inhibited ATPase activity in the hepatopancreas at low, medium, and high concentrations. At medium and high concentrations, ATPase activity in the heart, gills, and intestine was also significantly inhibited (Fig. 4C).

In comparison with the control group, Pb^2+^ exposure significantly inhibited NSE activity in the hepatopancreas at low, medium, and high concentrations. At high concentrations, it was also significantly inhibited in the gills and stomach. However, at low and medium concentrations, Pb^2+^ significantly promoted the NSE activity in the intestine (Fig. 4D). Similarly, Pb^2+^ significantly inhibited the POX activity in the heart, gills, and kidney at low, medium, and high concentrations, while significantly promoting this activity in the intestine at high concentrations (Fig. 4E). Compared with the control group, Pb^2+^ significantly inhibited SDH activity in the heart, hepatopancreas, and stomach at low, medium, and high concentrations (Fig. 4F).

Both heavy metals exerted inhibitory effects on various enzymes in the visceral organs of *M. anguillicaudatus,* showing clear tissue-specificity. Exposure to Cd^2+^ and Pb^2+^ mainly affected the functions of the heart, hepatopancreas, gills, and stomach of *M. anguillicaudatus*. Compared with Pb^2+^, Cd^2+^ exhibited a broader range of toxic effects across the six examined organs, which mainly manifested in the inhibition of ACP and ATPase activities in the kidney and stomach, whereas Pb^2+^ showed no significant effect on their activities. These findings suggest that both heavy metals cause extensive damage to the visceral organs of *M. anguillicaudatus*.

## Discussion

Enzyme histochemical staining technology is based on the reaction between enzymes and specific substrates to generate colored products, which indicates the presence and distribution of specific enzymes within cells or tissues. When combined with microscopic analysis, it allows for the accurate assessment of changes in enzyme activity under different conditions (Tomar & Bansal, 2023). Compared with traditional biochemical methods, enzyme histochemical staining does not require tissue homogenization and is relatively simple to perform. Additionally, frozen sections retain higher enzyme activity than non-frozen tissues, enabling multi-step staining analysis (Meier-Ruge & Bruder, 2008). Therefore, this study used this technology to detect the distribution of six metabolic and immune-related enzymes in the visceral organs of *M. anguillicaudatus* and to assess the effects of Cd^2+^ and Pb^2+^ on their activity.

Phosphatases are enzymes that catalyze the hydrolysis of various phosphate compounds. Depending on the optimal pH of their catalytic reactions, they can be divided into ACP and ALP (Bastiančić et al., 2023; Sang et al., 2023). ACP is a marker enzyme of lysosomes and plays an important role in intracellular digestion (Sang et al., 2023). ALP is related to the digestion, absorption, and transport of nutrients, such as glucose, lipids, calcium, and inorganic phosphate. Its activity reflects the organism’s digestion and absorption capacity (Tlak Gajger et al., 2024). Both enzymes effectively kill and digest pathogens, playing an important role in the immune defense of fish (Guo et al., 2023; Sang et al., 2023).

A previous study showed that the ACP activity of *Chelidonichthys lucerna* was significantly higher in the stomach and intestine (Bastiančić et al., 2023). Similarly, the present study showed that the ACP activity of *M. anguillicaudatus* was significantly higher in the stomach and intestine. However, unlike in *C. lucerna*, where ALP activity was concentrated in the intestine (Bastiančić et al., 2023), ALP activity in *M. anguillicaudatus* was the highest in the stomach, likely due to its strong nutrient absorption capacity. Di et al. (2021) reported that exposure to CdCl_2_ significantly increased the ACP activity in the serum of common carp (*Cyprinus carpio*), while significantly inhibiting ALP activity. Moreover, Onadje & Akalusi (2024) found that exposure of the African catfish (*Clarias gariepinus*) to Pb^2+^ caused ACP activity in the liver and kidney, as well as ALP activity in serum, to first increase and then decrease. Other studies have shown that long-term exposure to Cd^2+^ or Pb^2+^ pollution can cause significant tissue lesions and even necrosis in the hepatopancreas, gills, kidney, and intestine of fish (Jantawongsri et al., 2021; Di et al., 2021; Naz et al., 2021). Similarly, in this study, both Cd^2+^ and Pb^2+^ significantly inhibited ACP activity in the heart, hepatopancreas, gills, and intestine of *M. anguillicaudatus*, and the ALP activity in the hepatopancreas, gills, and stomach. Cd^2+^ also significantly inhibited the ACP activity of the kidney and stomach. Previous studies have attributed the inhibition of ACP and ALP activity by Cd^2+^ and Pb^2+^ to the destruction of the lysosomal structure and the reduction of enzyme synthesis due to the toxicity of these two heavy metals (George et al., 2011; Kulkarni et al., 2023). In this study, Cd^2+^ and Pb^2+^exhibited inhibitory effects on the ACP and ALP activities in multiple parts of *M. anguillicaudatus*, indicating that exposure to these heavy metal ions may impair the digestion and absorption capacity of *M. anguillicaudatus* and reduce its immune defense ability.

ATPase is a glycoprotein widely present in animal cells and organelle membranes. Its main function is to provide energy for transmembrane transport by hydrolyzing ATP and to maintain the ion gradient difference inside and outside the membrane (Gashkina, 2024; Li et al., 2023). Its activity is often used as a biomarker for evaluating heavy metal pollution (Atli et al., 2013; Da Silva & Martinez, 2014). To date, no studies have reported the distribution of ATPase in the various organs of fish. This study showed that the ATPase activity was significantly higher in the heart of *M. anguillicaudatus*, followed by the hepatopancreas and stomach. The heart is the power source of blood circulation, the hepatopancreas is an important metabolic and detoxification organ, and the stomach is the main digestive organ. Thus, the high ATPase activity in these organs may provide sufficient energy for *M. anguillicaudatus* to perform its normal functions.

Previous studies have reported that exposure to Cd^2+^ significantly reduces ATPase activity in the gills and kidneys of the freshwater teleost (*Prochilodus lineatus*) (Da Silva & Martinez, 2014). Similarly, exposure to Pb(NO_3_)_2_ can significantly reduce the ATPase activity in the gills of Indian catfish (*Heteropneustes fossilis*) (Mahapatra et al., 2022). Consistent with these findings, this study demonstrated that Cd^2+^ and Pb^2+^ significantly inhibited the ATPase activity in the heart, hepatopancreas, gills, and intestine of *M. anguillicaudatus*, and Cd^2+^ also inhibited ATPase activity in the kidney and stomach. Cd^2+^ exposure can interfere with the function of intracellular calcium and magnesium ion channels, destroy the balance of these ions, and inhibit ATPase activity (Yu et al., 2024). Moreover, Pb^2+^ can competitively bind to the Ca^2+^ binding sites of ATPase, thereby inhibiting ATPase activity (Dogan, Atli & Canli, 2015). The observed inhibition of Cd^2+^ and Pb^2+^ on ATPase activity in the heart, hepatopancreas, gills, and intestine of *M. anguillicaudatus* suggests the two heavy metal ions may inhibit blood circulation by affecting energy supply, impairing detoxification and respiration, and reducing the ability to absorb nutrients.

NSE is a carboxylic acid hydrolase that participates in the metabolism of fatty acids and glycerides in most vertebrates and plays an important role in the acquisition of energy substances in fish (Bastiančić et al., 2023; Tlak Gajger et al., 2024; Xie et al., 2016). Studies have shown that the inhibition of NSE enzyme activity is usually associated with various pollutants, such as pesticides, heavy metals, and surfactants (Kinareikina, Silivanova & Kyrov, 2024). In addition, esterases play an important role in the detoxification process of fish, and the activity of carboxylesterase in the hepatopancreas is closely related to the biodetoxification pathway of pesticides (Martínez-Morcillo et al., 2019). In vertebrates, NSE is mainly distributed in the liver, but is also present in other tissues, such as the digestive tract, brain, and muscles (Bastiančić et al., 2023; Martínez-Morcillo et al., 2019). This study showed that the activity of NSE in *M. anguillicaudatus* was highest in the hepatopancreas, consistent with its role as an important detoxification organ in fish.

Studies have shown that exposure to different concentrations of CdCl_2_ significantly inhibits acetylcholinesterase activity in the gills and liver of olive flounder (*Paralichthys olivaceus*) (Lee, Choi & Kim, 2022). Similarly, Li et al. (2007) found that NSE activity in the serum and blood of *Chlamys farreri* was significantly activated under Pb^2+^ stress. The findings of this study are consistent with these observations. Specifically, Cd^2+^ and Pb^2+^ significantly inhibited the activity of NSE in the hepatopancreas and stomach of *M. anguillicaudatus*, while Pb^2+^ significantly promoted the activity of NSE in the intestine. Previous studies have shown that Cd^2+^ exposure can cause oxidative stress, leading to structural damage of esterase protein, thereby inhibiting its activity (Xian et al., 2014). The Pb^2+^-induced activation of NSE in the *M. anguillicaudatus* intestine may present an anti-toxic defense mechanism caused by heavy metal stress (Oliva et al., 2019). Herein, the inhibitory effects of Cd^2+^ and Pb^2+^ on the activity of NSE in the hepatopancreas and stomach of *M. anguillicaudatus* indicate that these two heavy metal ions may damage the detoxification and lipid digestion ability of *M. anguillicaudatus*.

POX mainly exists in peroxisomes and is an important protective enzyme in the body. It can effectively defend against bacterial infection, eliminate harmful free radical substances, and prevent cells from oxidative damage (Sun et al., 2019). As a stress-sensitive enzyme, POX activity reflects the physiological status and environmental adaptability of the organism (Wagh, Osborne & Sivarajan, 2023). Studies have shown that POX is widely distributed in the intestine of *Cyprinus carpio* (Sun et al., 2019). In this study, POX activity was significantly higher in the kidney of *M. anguillicaudatus*, reflecting its key role as the main immune organ in fish. Studies have shown that the activity of antioxidant enzymes, such as GPx, significantly increases in the blood of Nile tilapia (*Oreochromis niloticus*) exposed to heavy metal contamination in river water (Hossain et al., 2021). Similarly, Pb^2+^ has been reported to significantly inhibit POX activity in the gills of *H. fossilis* (Mahapatra et al., 2022). Consistent with these findings, the present study revealed that both Cd^2+^ and Pb^2+^ significantly inhibited POX activity in the heart, kidney, and gills of *M. anguillicaudatus*. In contrast, Pb^2+^ significantly promoted POX activity in the intestine, which may be a transient stress response to resist metal toxicity and prevent tissue damage (Lebrun & Gismondi, 2020). Other studies have shown that Cd^2+^ reduces POX enzyme activity by binding with sulfhydryl (-SH) or replacing metal ions in the enzyme active site (Liu et al., 2022). In addition, oxidative stress induced by heavy metal exposure produces large amounts of peroxide free radicals, which further supress POX activity (Frías-Espericueta et al., 2022). The observed inhibition of POX activity by Cd^2+^ and Pb^2+^ in the heart, kidney, and gills of *M. anguillicaudatus* suggests that these two heavy metal ions could weaken the antioxidant capacity or immune defense function of *M. anguillicaudatus*.

SDH is an enzyme involved in carbon metabolism and cellular respiration, also known as complex II of the mitochondrial respiratory chain. It reflects the number and activity of mitochondria in the cells and is involved in key pathways, such as the tricarboxylic acid cycle and glucose metabolism (Bouillaud, 2023; Perumalsamy & Arumugam, 2013). SDH activity not only indicates the intensity of cellular energy metabolism but is also widely used as a biomarker for water pollution (Kadim & Risjani, 2022). However, to date, there have been no reports on the distribution of SDH in the various organs of fish. In this study, SDH activity in the heart of *M. anguillicaudatus* was significantly higher than in other tissues, followed by the hepatopancreas, reflecting the high energy demand associated with cardiac contraction and hepatopancreatic secretion. Studies have shown that SDH activity in the muscles of *Labeo rohita* was significantly reduced in rivers polluted by heavy metals, such as Cd^2+^ and Pb^2+^ (Das et al., 2018). In addition, Pb^2+^ can significantly inhibit SDH activity in the muscle tissue of *Pseudorasbora parva* (Marenkov et al., 2021). Similar to the above two species, both Cd^2+^ and Pb^2+^ significantly inhibited SDH activity in the heart, hepatopancreas, and stomach of *M. anguillicaudatus*. Cd^2+^ exposure is known to inhibit the mitochondrial electron transport chain, induce reactive oxygen species production, and inhibit SDH activity (Nair et al., 2013; Wang et al., 2004). The inhibition of SDH activity by Cd^2+^ and Pb^2+^ in *M. anguillicaudatus* indicated that these heavy metal ions could affect the energy metabolism of the heart, hepatopancreas, and stomach, thereby damaging blood circulation and its detoxification and digestion abilities.

## Conclusions

This study is the first to investigate the distribution of six important enzymes in *M. anguillicaudatus* organs and to examine the effects of Cd^2+^ and Pb^2+^ on their activities. The distribution of these enzymes were generally consistent with those reported in other species, but also exhibited species-specific characteristics. The stomach of *M. anguillicaudatus* showed strong digestive ability, the hepatopancreas had robust metabolic and detoxification capabilities, and the kidney demonstrated pronounce immune defense function. Both Cd^2+^ and Pb^2+^ were found to disrupt blood circulation by inhibiting the activity of various enzymes, impairing its detoxification and respiration, and inhibiting the digestion and absorption of nutrients by *M. anguillicaudatus*. Overall, this study enhances our understanding of the biological toxicity mechanisms of Cd^2+^ and Pb^2+^ and provides a theoretical basis for the clean and healthy breeding of *M. anguillicaudatus*.

##  Supplemental Information

10.7717/peerj.20547/supp-1 Supplemental Information 1 Raw data of distribution of six metabolic and immune-related enzymes in *Misgurnus anguillicaudatus.* ACP, acid phosphatase; ALP, alkaline phosphatase; ATPase, adenosine triphosphatase; ND, Not detectable; NSE, non-specific esterase; POX, peroxidase; SD, standard deviation; SDH, succinate dehydrogenase. Two-tailed independent Student’s *T* tests were employed for comparisons. Characters sharing the same letter in the same column indicate no significant difference.

10.7717/peerj.20547/supp-2 Supplemental Information 2 Measurement of the mean optical density of six metabolic and immune-related enzymes in the visceral organs of *Misgurnus anguillicaudatus* exposed to Cd^2+^ ACP, acid phosphatase; ALP, alkaline phosphatase; ATPase, adenosine triphosphatase; MOD, mean optical density; NSE, non-specific esterase; POX, peroxidase; SD, standard deviation; SDH, succinate dehydrogenase. Data expressed as mean ± SD, *n* = 9.

10.7717/peerj.20547/supp-3 Supplemental Information 3 Measurement of the mean optical density of six metabolic and immune-related enzymes in the visceral organs of *Misgurnus anguillicaudatus* exposed to Pb^2+^ ACP, acid phosphatase; ALP, alkaline phosphatase; ATPase, adenosine triphosphatase; MOD, mean optical density; NSE, non-specific esterase; POX, peroxidase; SD, standard deviation; SDH, succinate dehydrogenase. Data expressed as mean ± SD, *n* = 9.

10.7717/peerj.20547/supp-4 Supplemental Information 4 Statistical analysis of the effects of Cd^2+^ on the activities of metabolic and immune-related enzymes in *Misgurnus anguillicaudatus*. ACP, acid phosphatase; ALP, alkaline phosphatase; ATPase, adenosine triphosphatase; MOD, mean optical density; NSE, non-specific esterase; POX, peroxidase; SD, standard deviation; SDH, succinate dehydrogenase. Data expressed as mean ± SD, *n* = 9; *significant difference (*p* < 0.05); **extremely significant difference (*p* < 0.01).

10.7717/peerj.20547/supp-5 Supplemental Information 5 Statistical analysis of the effects of Pb^2+^ on the activities of metabolic and immune-related enzymes in *Misgurnus anguillicaudatus* ACP, acid phosphatase; ALP, alkaline phosphatase; ATPase, adenosine triphosphatase; MOD, mean optical density; NSE, non-specific esterase; POX, peroxidase; SD, standard deviation; SDH, succinate dehydrogenase. Data expressed as mean ± SD, *n* = 9; * significant difference (*p* < 0.05); ** extremely significant difference (*p* < 0.01).

10.7717/peerj.20547/supp-6 Supplemental Information 6 The arrive guidelines 2.0: author checklist

## Additional Information and Declarations

### Data Availability

The following information was supplied regarding data availability: The raw data are available in the Supplemental Files.
